# Autophagy and anti-inflammation ameliorate diabetic neuropathy with Rilmenidine

**DOI:** 10.1590/acb387823

**Published:** 2023-12-01

**Authors:** Mehmet Burak Yalçın, Ejder Saylav Bora, Adem Çakır, Sabiye Akbulut, Oytun Erbaş

**Affiliations:** 1 Bahcelievler Memorial Hospital – Department of Orthopedics and Traumatology – Istanbul – Turkey.; 2 Izmir Ataturk Research and Training Hospital – Department of Emergency Medicine – Izmir – Turkey.; 3 Canakkale Mehmet Akif Ersoy State SBU Kartal Kosuyolu Training and Research Hospital – Department of Emergency Medicine – Canakkale – Turkey.; 4 SBU Kartal Kosuyolu Training and Research – Hospital Department of Gastroenterology – Istanbul – Turkey.; 5 Demiroğlu Bilim University – Faculty of Medicine – Department of Physiology – Istanbul – Turkey.

**Keywords:** Diabetic Neuropathies, Rilmenidine, Electromyography, Autophagy

## Abstract

**Purpose:**

To evaluate the neuroprotective effects of Rilmenidine on diabetic peripheral neuropathy (DPN) in a rat model of diabetes induced by streptozotocin (STZ).

**Methods:**

STZ (60 mg/kg) was administered to adult Sprague-Dawley rats to induce diabetes. On the 30th day after STZ administration, electromyography (EMG) and motor function tests confirmed the presence of DPN. Group 1: Control (n = 10), Group 2: DM + 0.1 mg/kg Rilmenidine (n = 10), and Group 3: DM + 0.2 mg/kg Rilmenidine (n = 10) were administered via oral lavage for four weeks. EMG, motor function test, biochemical analysis, and histological and immunohistochemical analysis of sciatic nerves were then performed.

**Results:**

The administration of Rilmenidine to diabetic rats substantially reduced sciatic nerve inflammation and fibrosis and prevented electrophysiological alterations. Immunohistochemistry of sciatic nerves from saline-treated rats revealed increased perineural thickness, HMGB-1, tumor necrosis factor-α, and a decrease in nerve growth factor (NGF), LC-3. In contrast, Rilmendine significantly inhibited inflammation markers and prevented the reduction in NGF expression. In addition, Rilmenidine significantly decreased malondialdehyde and increased diabetic rats’ total antioxidative capacity.

**Conclusions:**

The findings of this study suggest that Rilmenidine may have therapeutic effects on DNP by modulating antioxidant and autophagic pathways.

## Introduction

Diabetic peripheral neuropathy (DPN), a prevalent complication of diabetes, affects approximately 50% of individuals with diabetes^1^. It is distinguished by gradual nerve deterioration following a specific pattern, resulting in symptoms such as pain, diminished sensation, and eventually complete loss of sensation in the hands and feet. Although high blood sugar levels are considered the leading cause of DPN, the exact mechanisms involved are not entirely understood. Several theories explain how chronic hyperglycemia leads to nerve damage, including metabolic irregularities, neurotrophic factors, immunological mechanisms alterations, and increased production of reactive oxygen species^2^^,^ 3. These metabolic abnormalities can cause damage to nerve cells, leading to the development and progression of DPN^4^^,^ 5.

A progressive loss of nerve fibers characterizes DPN and is the primary cause of numbness and amputation risk in the extremities^6^. Electrophysiological examinations, including electromyography (EMG), are the most accurate way of diagnosing DPN^7^^,^ 8. Diabetic peripheral neuropathy is associated with changes in sensory and motor nerve conduction velocity (NCV), including a decrease in amplitude and an increase in the temporal dispersion of compound muscle action potentials (CMAP). Axonal loss, demyelination, decreased NCV, decreased CMAP amplitude, and extended CMAP latency have been observed in the sciatic nerves of diabetic rats. Demyelination and decreased CMAP amplitude have also been monitored. Nerve growth factor (NGF) is a neuroprotective agent that restores function, improving DPN’s treatment mechanisms^9^^,^ 10.

Furthermore, the utilization of inclined plane testing plays a crucial role in the pathophysiologic investigation and advancement of drug treatments for neuropathy. This strategy establishes applicable methods and assesses muscle weakness, particularly in rat models with spinal cord injuries. In addition, both human and animal neuropathy patients frequently exhibit motor and sensory impairments. The inclined plane test is a quantitative and objective method for evaluating motor function impairment^11^. Therefore, following the onset of DPN, we conducted tests on rats on an inclined plane and analyzed the results.

Rilmenidine, an imidazoline compound, has a binding affinity for imidazoline receptors, such as the I1 and I2, but it is relatively weak compared to its affinity for α2-adrenergic receptors^12^. Therefore, the imidazoline receptor binding affinity of Rilmenidine is considered secondary to its direct action on α2-adrenergic receptors. By binding and activating imidazoline receptor and α2-adrenergic receptors (particularly the α2A subtype), Rilmenidine becomes essential in regulating blood pressure, sympathoinhibition, and neuroprotective effects 12.

The existence of three primary classes of imidazoline receptors is widely acknowledged. The I(1) imidazoline receptor facilitates sympatholytic inhibition to reduce blood pressure. An essential monoamine oxidase allosteric binding site is the I(2) receptor. Lastly, the I(3) receptor regulates insulin secretion from pancreatic beta cells^13^.

On the other hand, Rilmendine has demonstrated the ability to regulate autophagy, which is the mechanism by which cells eliminate and recycle damaged or dysfunctional components^14^. Autophagy plays a crucial role in maintaining cellular balance and is implicated in the pathogenesis of several diseases when dysregulated.

In this investigation, we explored the potential effect of Rilmendine on DPN. Our study of diabetic rats involved EMG, histopathological examination, and immunohistochemistry. In addition, we evaluated the impact of Rilmenidine on DPN using clinical observation techniques, such as inclined plane testing.

## Methods

### Study design

This was an experimental study for evaluating the Rilmenidine effect on nerve injury.

### Settings

#### Animals

Forty male and adult Wistar rats weighing 200 and 210 grams were used for the study. The rats were housed in enclosures and kept in normal conditions, with a 12-hour light and 12-hour dark cycle and average temperature of 22°C. Throughout the investigation, they had access to a conventional pellet diet and tap water. The Institutional Animal Care and Ethical Committee of the University of Science authorized the study protocol (Ethical Number: 923055817). Unless otherwise specified, all chemicals used in the study were obtained from Sigma-Aldrich Inc.

#### Experimental protocol

Thirty rats were injected intraperitoneally (IP) with 60 mg/kg of streptozotocin (STZ) in 0.9% NaCl, pH 4, adjusted with 0.2M sodium citrate to induce diabetes. Ten additional rats (group 1) served as a control group, receiving no chemical treatment. The glucose levels of the control group fell below 120 mg/dL and were within the normal range. After 24 hours, diabetes was confirmed by measuring blood glucose levels with glucose oxidase reagent strips manufactured by Boehringer-Mannheim in Indianapolis. Diabetic rats had blood glucose levels of at least 250 mg/dL. Group 2 (n = 10) received 0.1 mg/kg/day of Rilmenidine (Hyperium 1 mg, Servier) via oral gavage. Group 3 (n = 10) was administered 0.2 mg/kg/day of Rilmenidine by oral gavage for four weeks. The rats were evaluated using EMG and inclined plane testing following the investigation at the end of the study.

All animals were humanely euthanized after the investigation using cervical dislocation under anesthesia. Anesthesia was administered using 100 mg/kg of ketamine (Ketasol, Richterpharma AG Austria) and 50 mg/kg of xylazine (Rompun, Bayer, Germany). In addition, the heart was punctured to acquire blood samples for biochemical analysis, and samples of the sciatic nerve were obtained for immunohistochemistry and biochemical analysis.

### Data sources

#### Electromyography

For EMG testing, the right sciatic nerve of each rat was stimulated three times with supra-maximal intensity (10 V) and frequency (1 Hz) using a bipolar subcutaneous needle stimulation electrode (BIOPAC Systems, Inc., Santa Barbara, CA, United States of America) inserted into the Achilles tendon. The stimulation frequency range was between 0.5 and 5,000 Hz, with a 40-kHz/sec sampling rate. To record the compound muscle action potentials (CMAPs) and variations in motor NCV, unipolar needle electrodes were inserted into the 2-3 interosseous muscles to record the CMAPs and variations in motor NCV. Using the software Biopac Student Lab Pro version 3.6.7 (BIOPAC Systems, Inc.), the distal latency, duration, and amplitude parameters of CMAP were evaluated. During EMG recordings, each rat’s rectal temperature was monitored using a rectal sensor (HP Viridia 24-C; Hewlett-Packard Company, Palo Alto, CA, United States of America) and maintained at approximately 36°C to 37°C utilizing a heating pad.

#### Examining the sciatic nerve histopathologically

Hematoxylin and eosin were used to stain sections of the sciatic nerve (4 uM) that had been formalin fixed. Then, the perineural thickness of the sciatic nerve was determined using an Olympus C-5050 digital camera attached to an Olympus BX51 microscope.

#### Inclined plane test

To evaluate the motor performance of rats one month after STZ induction, we modified a sliding apparatus based on previous research^15^^,^ 16. The device for gliding was comprised of a 50 cm × 30 cm stainless steel plane. We determined the angle at which a rat’s limb could slide while maintaining body position. The evaluation was conducted three times for each head position, and the average of the results was calculated. There was a 1-minute gap between trials.

#### Assessment of lipid peroxidation

To determine lipid peroxidation, the researchers measured the plasma samples’ malondialdehyde (MDA) levels. This level was accomplished using the thiobarbituric acid reactive substance (TBARS) method. The plasma samples were treated with trichloroacetic acid and TBARS reagent, mixed, and incubated at 100°C for 60 minutes. After 20 minutes of chilling at 3,000 rpm and centrifugation, the absorbance of the supernatant was measured at 535 nm. The MDA levels were calibrated using tetraethoxypropane and expressed as nM.

#### Analysis of sciatic nerve biochemistry

Following decapitation, the entire sciatic nerves were extracted and frozen at -20°C until biochemical analysis could be performed. Nerves were homogenized in 5-vol phosphate-buffered saline (pH = 7.4) using a glass homogenizer and centrifuged at 5,000 g for 15 minutes to prepare samples for research. The supernatant was collected, and the Bradford method determined the total protein concentration using bovine serum albumin as a standard 17.

The tumor necrosis factor (TNF)-a, NGF, LC-3, and HMGB-1 levels in sciatic nerve tissue were determined using rat-specific enzyme-linked immunosorbent assay (ELISA) reagents. The supernatants from the sciatic nerve samples were used for the measurement, and the assays were performed according to the manufacturer’s instructions. Each sample’s absorbance was measured in triplicate using a microplate reader: Thermo Fisher Scientific Laboratory Equipment, MultiscanGo, New Hampshire, United States of America.

### Statistical methods

Data were analyzed with Statistical Package for the Social Sciences program version 26.0. Number, percentage, mean, standard deviation, median, minimum, and maximum were used to present descriptive data. The conformity of the data to the normal distribution was evaluated with the Kolmogorov-Smirnov’s Test. Continuous variables with normal distribution in univariate analysis were expressed as mean ± standard deviation, and median (IQR) values were used for data that did not show normal distribution. Pearson χ^2^ test was used in the analysis of categorical variables. Fisher’s exact test was used in the presence of less than five populations in categorical variables. A T-test was used to compare two independent quantitative data. Parametric variables between groups were compared using the Student’s t-test. For the one-way analysis of variance test, the Bonferroni type was chosen. A p of less than 0.05 was considered statistically significant, whereas a p of less than 0.001 was considered highly effective.

## Results

### Electromyography

**Figure 1:**
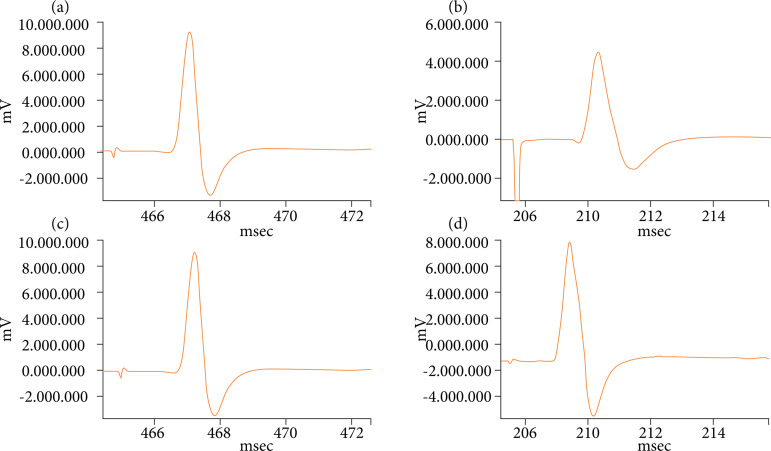
Samples of compound muscle action potential from the sciatic nerve of **(a)** control group electromyography, **(b)** diabetic and saline treatment group electromyography, **(c)** diabetic and 0.1 mg/kg Rilmenidine treatment group electromyography, **(d)** diabetic and 0.2 mg/kg Rilmenidine treatment group electromyography.

Source: Elaborated by the authors.

Regarding CMAP amplitude, the control group exhibited a mean value of 13.3 ± 0.5 mV. In contrast, the diabetes + saline treatment group showed a significantly lower CMAP amplitude of 8.1 ± 0.2 mV (p < 0.05). However, the two Rilmenidine-treated groups demonstrated improvement in CMAP amplitudes compared to the diabetes + saline group. Specifically, the diabetes + 0.1 mg/kg Rilmenidine group had a CMAP amplitude of 12.2 ± 0.5 mV, while the diabetes + 0.2 mg/kg Rilmenidine group exhibited a slightly higher value of 12.9 ± 0.3 mV. However, these differences were not statistically significant when compared to the control group or each other (Fig. 1, Table 1).

Regarding distal latency, the control group had a mean value of 1.28 ± 0.08 ms. In contrast, the diabetes + saline treatment group showed a significantly higher distal latency of 2.05 ± 0.1 ms (p < 0.05). In this parameter, both Rilmenidine-treated groups demonstrated betters results compared to the diabetes + saline group. The diabetes + 0.1 mg/kg Rilmenidine group had a distal latency of 1.48 ± 0.2 ms, while the diabetes + 0.2 mg/kg Rilmenidine group had a slightly lower value of 1.35 ± 0.1 ms. These differences were statistically significant (p < 0.05) when compared to the diabetes + saline group, indicating a beneficial effect of Rilmenidine treatment on distal latency (Fig. 1, Table 1).

**Table 1:** Results of the analyses performed with the electromyography Data are expressed as mean ± standard error of the mean.

	Control group	Diabetes and saline treatment	Diabetes and 0.1 mg/kg Rilmenidine treatment	Diabetes and 0.2 mg/kg Rilmenidine treatment
Compound muscle action potential Amplitude (mV)	13.3 ± 0.5	8.1 ± 0.2*	12.2 ± 0.5#	12.9 ± 0.3#
Distal latency (ms)	1.28 ± 0.08	2.05 ± 0.1*	1.48 ± 0.2#	1.35 ± 0.1#

* p < 0.05 (different from control);

# p < 0.05 (different from diabetes + saline).

### Inclined plane test

The mean maximal angle of the inclined plane test for the control group was 85.1 ± 3.3 degrees (standard deviation). Nevertheless, the diabetes + saline treatment group had a substantially reduced maximum angle of 53.7 ± 4.1 degrees (p = 0.001) than the control group. In contrast, both Rilmenidine-treated groups outperformed diabetes + saline group on the inclined plane test. The diabetes + 0.1 mg/kg Rilmenidine group attained a maximal angle of 81.9 ± 6.8 degrees, whereas the diabetes + 0.2 mg/kg Rilmenidine group obtained a rise of 82.5 ± 2.4 degrees. These differences were statistically significant (p = 0.05) compared to the diabetes + saline group, suggesting that treatment with Rilmenidine may enhance motor function and mobility in people with diabetes (Table 2).

**Table 2:** Inclined plane test power analysis results. Data are expressed as mean ± standard error of the mean.

	Control group	Diabetes and saline treatment	Diabetes and 0.1 mg/kg Rilmenidine treatment	Diabetes and 0.2 mg/kg Rilmenidine treatment
Maximum angle of inclined plane test (degree)	85.1 ± 3.3	53.7 ± 4.1*	81.9 ± 6.8#	82.5 ± 2.4#
Plasma glucose (mg/dL)	98.3 ± 6.5	415.2 ± 11.6*	367.1 ± 13.5#	315.4 ± 9.1#

* p < 0.001 (different from control);

# p < 0.05 (different from diabetes + saline).

### Plasma glucose levels

Regarding plasma glucose levels (Table 2), the control group had a mean value of 98.3 ± 6.5 mg/dL. In contrast, the diabetes + saline treatment group exhibited significantly higher plasma glucose levels of 415.2 ± 11.6 mg/dL (p < 0.001) compared to the control group. However, both Rilmenidine-treated groups showed lower plasma glucose levels than the diabetes + saline group. The diabetes + 0.1 mg/kg Rilmenidine group had plasma glucose levels of 367.1 ± 13.5 mg/dL, while the diabetes + 0.2 mg/kg Rilmenidine group had a further reduction, with 315.4 ± 9.1 mg/dL. These differences were statistically significant (p < 0.05) compared to the diabetes + saline group, indicating that Rilmenidine treatment may positively affect glycemic control in individuals with diabetes.

### Histological results

**Figure 2:**
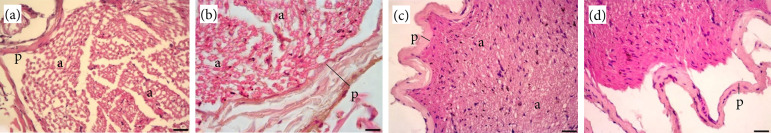
The histological sections of the sciatic nerve. Hematoxylin and eosin staining (x 40 magnification). **(a)** Control group p: perineurium and perineural thickness, a: axon. **(b)** Diabetes mellitus and the saline-treated group was shown increased perineural thickness. **(c)** Diabetes mellitus and 0.1 mg/kg Rilmenidine treatment group was shown decreased perineural thickness. **(d)** Diabetes mellitus and 0.2 mg/kg Rilmenidine treatment group showed decreased perineural thickness (scale bar = 20 µM).

Source: Elaborated by the authors.

Histological sections of the sciatic nerve were stained with hematoxylin and eosin (H&E) to evaluate the effects of different interventions on the perineurium and perineural thickness. The normative group (Fig. 2a) showed normal perineurium and perineural thickness, with clearly visible axons (a). However, in the diabetes mellitus and saline-treated group (Fig. 2b), an increase in perineural thickness was observed compared to the control group (Fig. 2).

### Perineural thickness in the sciatic nerve

In the diabetes mellitus and Rilmenidine-treated groups, a dose-dependent decrease in perineural thickness was observed. A significant reduction in perineural thickness was evident in the diabetes mellitus and 0.1 mg/kg Rilmenidine treatment group (Fig. 2c) compared to the diabetes mellitus and saline-treated group. Furthermore, in the diabetes mellitus and 0.2 mg/kgRilmenidine treatment group (Fig. 2d), perineural thickness was further decreased compared to the diabetes mellitus and saline-treated groups and the diabetes mellitus and 0.1 mg/kg rilmenidine treatment group (Fig. 2).

The perineural thickness in the diabetes + saline treatment group was markedly more significant than in the control group (3.8 ± 0.1 m). However, the perineural thickness was diminished in both Rilmenidine-treated groups: diabetes + 0.1 mg/kg rilmenidine (10.8 ± 1.1 m) and diabetes + 0.2 mg/kg Rilmenidine (6.3 ± 0.8 m). These differences were statistically significant compared to the diabetes + saline group (p < 0.05 and p < 0.01, respectively), indicating a potential beneficial effect of Rilmenidine treatment on perineural thickness (Table 3).

**Table 3:** Biochemical blood and tissue analysis. Data are expressed as mean ± standard deviation.

Biochemical blood and tissue analysis	Control group	Diabetes and saline treatment	Diabetes and 0.1 mg/kg Rilmenidine treatment	Diabetes and 0.2 mg/kg Rilmenidine treatment
Perineural thickness (µM)	3.8 ± 0.1	15.4 ± 2.9*	10.8 ± 1.1#	6.3 ± 0.8##
Sciatic nerve tumor necrosis factor-α level (pg/mg)	26.2 ± 2.08	48.5 ± 1.7*	33.1 ± 0.9#	30.3 ± 0.5#
Sciatic nerve growth factor level (pg/mg)	75.9 ± 5.7	44.5 ± 2.1*	56.4 ± 1.5#	60.8 ± 4.06#
Sciatic nerve LC-3 level (pg/mg)	38.5 ± 1.1	29.2 ± 0.8*	68.4 ± 2.3#	82.7 ± 1.9#
Sciatic nerve HMGB-1 level (pg/mg)	4.01 ± 0.07	9.2 ± 0.1*	5.3 ± 0.08#	5.2 ± 0.1#
Plasma malondialdehyde (nM)	50.8 ± 1.5	168.5 ± 7.3**	75.1 ± 6.6#	68.9 ± 5.7##

* p < 0.01,

* p < 0.001 (different from control);

# p < 0.05,

## p < 0.01 (different from diabetes + saline).

### Antioxidant anti-inflammatory parameters and nerve growth factor immunoexpression

The diabetes + saline treatment group exhibited significantly higher levels of TNF-α in the sciatic nerve (48.5 ± 1.7 pg/mg) compared to the control group (26.2 ± 2.08 pg/mg) (p < 0.001). In contrast, both Rilmenidine-treated groups showed reduced TNF-α levels: diabetes + 0.1 mg/kg Rilmenidine group (33.1 ± 0.9 pg/mg), and diabetes + 0.2 mg/kg Rilmenidine group (30.3 ± 0.5 pg/mg). These differences were statistically significant compared to the diabetes + saline group (p < 0.05), indicating a potential anti-inflammatory effect of Rilmenidine treatment on TNF-α levels in the sciatic nerve (Table 3).

The diabetes + saline treatment group exhibited significantly lower levels of NGF in the sciatic nerve (44.5 ± 2.1 pg/mg) compared to the control group (75.9 ± 5.7 pg/mg) (p < 0.01). However, both Rilmenidine-treated groups showed increased NGF levels: the diabetes + 0.1 mg/kg Rilmenidine group (56.4 ± 1.5 pg/mg) and the diabetes + 0.2 mg/kg Rilmenidine group (60.8 ± 4.06 pg/mg). These differences were statistically significant compared to the diabetes + saline group (p < 0.05), indicating a potential neurotrophic effect of Rilmenidine treatment on NGF levels in the sciatic nerve (Table 3).

Levels of LC-3 in the sciatic nerve were significantly lower in the diabetes + saline treatment group (29.2 ± 0.8 pg/mg) compared to the control group (38.5 ± 1.1 pg/mg) (p < 0.001). However, both Rilmenidine-treated groups showed increased LC-3 levels: the diabetes + 0.1 mg/kg Rilmenidine group (68.4 ± 2.3 pg/mg), and the diabetes + 0.2 mg/kg Rilmenidine group (82.7 ± 1.9 pg/mg). These differences were statistically significant compared to the diabetes + saline group (p < 0.001), suggesting a potential role of Rilmenidine in enhancing autophagy activity in the sciatic nerve (Table 3).

The diabetes + saline treatment group had significantly higher levels of HMGB-1 in the sciatic nerve (9.2 ± 0.1 pg/mg)compared to the control group (4.01 ± 0.07 pg/mg) (p < 0.001). However, both Rilmenidine-treated groups showed decreased HMGB-1 levels: diabetes + 0.1 mg/kg Rilmenidine group (5.3 ± 0.08 pg/mg) and diabetes + 0.2 mg/kg Rilmenidine group (5.2 ± 0.1 pg/mg). These differences were statistically significant compared to the diabetes + saline group (p < 0.05), indicating a potential modulatory effect of Rilmenidine treatment on HMGB-1 levels in the sciatic nerve (Table 3).

Plasma MDA concentrations in the diabetes + saline treatment group were significantly greater than in the control group (50.8 ± 1.5 nM; p = 0.001). However, plasma MDA levels were reduced in both Rilmenidine-treated groups: diabetes + 0.1 mg/kg Rilmenidine group (75.1± 6.6 nM), and diabetes + 0.2 mg/kg Rilmenidine group (68.9 ± 5.7 nM). Compared to the diabetes + saline group, these differences were statistically significant (p < 0.05 and p < 0.01, respectively), indicating a potential antioxidative effect of Rilmenidine treatment on plasma MDA levels (Table 3).

## Discussion

Diabetes has become a disease that is mainly feared for its complications. The most common of these complications is DPN. Inadequate reactive oxygen species (ROS) production due to oxidative stress, advanced glycation end products (AGEs), and inflammatory pathways comprise the mechanism of diabetic neuropathy^1^.

Histological examination of sciatic nerve sections revealed that Rilmenidine treatment resulted in a dose-dependent reduction in perineural thickness compared to the diabetic and saline-treated group. This result indicates that Rilmenidine may have a protective effect on the perineurium. This connective tissue layer surrounds the nerve fibers and plays a critical role in maintaining nerve integrity. The observed reduction in perineural thickness suggests that Rilmenidine treatment may attenuate the pathological changes associated with DPN, potentially preserving nerve function. Similarly, a study by Maiese et al. observed that the agents acting like imidazole receptors show neuroprotective effects in cerebral ischemic patients^18^.

The leading cause of diabetic neuropathy is the death of Schwann cells. In immunohistochemical analysis of the sciatic nerves of diabetic rats, we can see the death of Schwann cells and the regression of NGF, which is secreted by Schwann cells. It is known that NGF affects the restructuring and growth of the nerve and the modulation of autophagy. This situation increases NGF, and it probably increases the efficiency of autophagy^14^.

In this study, DPN was diagnosed using EMG. Electrophysiological examinations, including EMG and nerve cell biopsies, are valuable tools in diagnosing neuropathy^19^. Both diabetic patients and rat models exhibited axonal loss, demyelination, decreased NCV, decreased CMAP amplitude, and long CMAP latency in the sciatic nerve^20^. In addition, EMG analysis revealed that Rilmenidine treatment enhanced muscle activity compared to the diabetic and saline-treated groups. The abnormal EMG patterns observed in the diabetic and saline-treated groups, indicative of impaired muscle function, were partially restored in the diabetic and Rilmenidine-treated groups. EMG results suggest that Rilmenidine may benefit muscle activity, potentially contributing to ameliorating DPN-related muscle dysfunction. In addition, the sciatic nerve of diabetic rats exhibits histological signs of neuropathy, such as axonal degeneration, myelin distention, and perineurial fibrosis^16^.

Autophagy is a cellular process that degrades and recovers damaged or dysfunctional components, contributing to cellular homeostasis. Dysregulation of autophagy has been implicated in the pathogenesis of various diseases, including DPN. LC-3 is a marker commonly used to monitor autophagy, a cellular process involved in the degradation and recycling of cellular components^21^. Zatyka et al., in 2020, described the role of autophagy in neurodegenerative diseases^22^. In addition, Zhang et al.’s 2013 study shows that LC-3 significantly increases in patients with neuropathy in a model with nerve ligation. LC-3 again shows the role of autophagy in nerve damage. Rilmenidine was reported for its ability to induce autophagy and attenuate the toxicity of mutant huntingtin in a mouse model of Huntington disease^23^. In a study by Mercer et al., Rilmenidine similarly shows a modulatory effect in autophagy by inducing LC-3 activity^24^. This study observed that LC-3 value in tissue samples taken from the nerve increased in DMP patients and decreased with Rilmenidine. The ability of Rilmenidine to modulate autophagy promotes the clearance of damaged cellular components, thus attenuating neuropathic damage.

Rilmenidine acts on the sympathetic nervous system via Alpha-2 adrenergic receptors. Activation of these receptors may modulate inflammation-related events by regulating sympathetic activity and reducing the release of norepinephrine. Moreover, some studies have shown that Rilmenidine inhibits the production of pro-inflammatory cytokines (such as TNF-α and interleukin-6)^25^. On the other hand, it increases GABA, which is the primary inhibitory neurotransmitter. This inhibition can affect neurotransmitter movement in the inflammatory process^26^.

In this study, inflammatory parameters TNF-α and HMGB-1 were increased in diabetic rats due to the nature of neuroinflammation. Although HMGB-1 acts as a danger signal in the body released by damaged or dying cells and triggers inflammation, the primary function of TNF-α is to initiate and regulate inflammation. The significant decrease in these values in 0.1 and 0.2 mg/kg Rilmenidine use compared to the diabetes mellitus + saline group shows that the anti-inflammatory effect of Rilmenidine is not dose-dependent in terms of inflammation.

However, in MDA results, which is a marker of oxidative damage, 0.2 mg/kg Rilmenidine showed a more substantial effect on the diabetes mellitus + saline group than 0.1 mg/kg, indicating that the antioxidant effect of high doses of Rilmenidine is stronger than lower doses. In the study conducted by Salman et al. in 2011, the antioxidant effect of Rilmendine was emphasized^27^, and similar results were found^28^. One of the complications of diabetes mellitus is impaired filtration and microalbuminuria due to renal damage. Studies have shown that Rilmenidine decreases microalbuminuria and indirectly prevents the increase in lipid peroxidation, which also shows that it reduces oxidative damage at this point^29^^,^ 30.

NGF is essential for forming, maintaining, and restoring the peripheral nervous system. In some cases, there is a decrease in NGF levels, which may contribute to developing and progressing neuropathic symptoms. NGF deficiency can lead to impaired nerve regeneration, increased apoptosis, and decreased nerve fiber density^31^. NGF also has indirect effects on inflammation and neuroprotection and direct effects on nerve regeneration. NGF can modulate the production of pro-inflammatory cytokines and chemokines involved in the pathogenesis of DPN, such as TNF-αlpha and interleukin-6^32^^,^ 33. By reducing inflammation, NGF may help mitigate nerve damage and promote healing in DPN. Furthermore, NGF has been shown to have trophic effects on sensory neurons, promoting their survival and preventing apoptosis. Similar to our study with Rilmenidine, NGF can also enhance the function of Schwann cells, which play a crucial role in nerve regeneration and myelination^32^.

Rilmenidine has been also found to have an impact on glucose regulation. The favorable effect of Rilmenidine on an animal model of insulin resistance suggests its ability to decrease sympathetic system activity^32^. In addition, in a study by Luca et al. in 2000, fasting blood glucose levels and postprandial glucose levels tended to decrease with rilmenidine^34^. Consistent with the literature, this study also observed a decrease in glucose levels following the administration of Rilmenidine.

### Limitation

This study was conducted using an animal model; additional research is required to ascertain the efficacy and safety of Rilmenidine in humans. Additionally, the precise molecular mechanisms underlying the effects of Rilmenidine on autophagy and inflammation require further investigation. On the other hand, in using Rilmenidine, different doses can be tried in this direction. The long-term effects of Rilmenidine on diabetic rats can be tried. Prospective randomized studies can be conducted on the EMG outcomes of diabetic people.

## Conclusion

Rilmenidine holds promise as a potential therapeutic agent for treating DPN by modulating autophagy and exerting anti-inflammatory effects. Rilmenidine may help mitigate nerve damage, improve nerve function, and alleviate neuropathic symptoms. Further investigations will shed more light on the precise mechanisms of action and clinical potential of Rilmenidine, ultimately offering new avenues for managing DPN.

## Data Availability

All data for the study are presented in the published article.
